# Regeneration of Hair Cells: Making Sense of All the Noise

**DOI:** 10.3390/ph4060848

**Published:** 2011-06-17

**Authors:** Benjamin Kopecky, Bernd Fritzsch

**Affiliations:** 1 Department of Biology, University of Iowa, Iowa City, IA, 52242, USA; 2 Medical Scientist Training Program, Carver College of Medicine, University of Iowa, Iowa City, IA, 52242, USA

**Keywords:** iPSCs, miRNAs, stem cells, hair cells, regeneration, EPI-NCSCs

## Abstract

Hearing loss affects hundreds of millions of people worldwide by dampening or cutting off their auditory connection to the world. Current treatments for sensorineural hearing loss (SNHL) with cochlear implants are not perfect, leaving regenerative medicine as the logical avenue to a perfect cure. Multiple routes to regeneration of damaged hair cells have been proposed and are actively pursued. Each route not only requires a keen understanding of the molecular basis of ear development but also faces the practical limitations of stem cell regulation in the delicate inner ear where topology of cell distribution is essential. Improvements in our molecular understanding of the minimal essential genes necessary for hair cell formation and recent advances in stem cell manipulation, such as seen with inducible pluripotent stem cells (iPSCs) and epidermal neural crest stem cells (EPI-NCSCs), have opened new possibilities to advance research in translational stem cell therapies for individuals with hearing loss. Despite this, more detailed network maps of gene expression are needed, including an appreciation for the roles of microRNAs (miRs), key regulators of transcriptional gene networks. To harness the true potential of stem cells for hair cell regeneration, basic science and clinical medicine must work together to expedite the transition from bench to bedside by elucidating the full mechanisms of inner ear hair cell development, including a focus on the role of miRs, and adapting this knowledge safely and efficiently to stem cell technologies.

## 1. Introduction

Sensorineural hearing loss (SNHL) afflicts over 278 million individuals worldwide and nearly half of all individuals over the age of 65 [1-3]. This multifactorial disease results from gene mutations, ototoxic drugs, environmental insults, or aging, and is often irreversible. Hearing loss imparts a substantial financial cost to society and bears an emotional and quality of life burden to the affected individual along with his/her family [1,4,5]. Current treatments with cochlear implants require an invasive surgical procedure without faithfully recapitulating normal hearing. Cochlear implants do not represent a perfect cure but rather a crutch to obtain some hearing capability [1, 6-12]. Prevention of hearing loss and regenerative medicine are two alternative pathways toward a possible perfect cure [13-15]. While prevention of hearing loss through protection of hair cells is ideal, it is not always possible [16-19], leaving regenerative medicine the sole *ex post facto* option for a permanent and restorative treatment of hearing loss. However, regeneration of damaged sensory epithelia in the mammalian inner ear is complicated as differentiated adult inner ear neurosensory tissue cannot re-enter the cell cycle and be used to replace neighboring cells, as is spontaneously accomplished in non-mammalian systems. This absence of proliferative capacity necessitates the use of exogenous stem cells as a medium for hair cell restoration, unless possibilities are discovered to jumpstart adult neurosensory epithelia proliferation.

The organ of Corti is a highly specialized sense organ with polarized and highly ordered inner and outer hair cells positioned in a stereotypic pattern on the basilar membrane surrounded by multiple, specialized supporting cells. Inner hair cells are innervated by approximately 95% of all sensory neurons and play the major role in sound perception while outer hair cells serve to fine-tune the hearing sensitivity. SNHL results primarily from damage to the inner hair cells in the organ of Corti. Regenerative treatment must therefore be aimed at replacing damaged organ of Corti inner hair cells; this can be done by either regenerating an entirely new organ of Corti or by replacing only the damaged hair cells, on a one-by-one basis. As inner hair cells die, morphologic changes occur to the organ of Corti including eventual loss of afferent neurons, making replacement of lost hair cells on a one-by-one basis problematic, in particular, years after loss of hair cells and subsequent organ of Corti dedifferentiation. In the absence of hair cells and key neurotrophins, originating from organ of Corti cells, spiral ganglion neurons are progressively lost [20,21]. The organ of Corti's central projections [20] form a complex tonotopic map that may not be capable of being faithfully recapitulated in a regenerated organ of Corti as brain plasticity declines with age [22-26]. If central projections cannot be retained in patients suffering from SNHL for years, not only must the individual hair cells be reformed, but so too must the neurons. In such cases, regeneration of the entire organ of Corti (with its delaminating sensory neurons) may provide the only feasible therapeutic option. This approach requires an initial jumpstart of the organ of Corti developmental process using stem cells transplanted into the cochlea, after which the context within the cochlea may suffice to activate neurosensory cell developmental programs to form a replacement organ of Corti. However, it is unlikely that an adult cochlea will provide the contextual environment needed as developmental signaling has long been turned off [27]. Therefore, stepwise cell fate restriction of transplanted stem cells using molecular signals required for organ of Corti induction, specification, and neurosensory cell formation is needed. Alternatively, the development program for the organ of Corti must be re-initiated to reform through proliferation and differentiation the lost organ. Additionally, applications of neurotrophic factors may be needed to facilitate a functional connection between the brain and the inner ear.

In patients suffering from short-term hearing loss, regeneration of damaged portions of the organ of Corti on a one-by-one basis may be possible as sensory neurons have not undergone apoptosis and the organ of Corti has not dedifferentiated. This therapeutic option requires a similar molecular understanding of neurosensory cell development but in addition requires exact targeting of prosensory cells to regions of damaged sensory epithelia while retaining proper orientation, structure, and functionality of healthy neighboring tissue. Transdifferentiation of neighboring cells cannot be used in mammals as they would deplete supporting cell populations as supporting cells are currently unable to re-enter the cell cycle [28-38]. *In vitro* attempts using stem cells have been initiated to reconstitute hair cells in the murine system and have been shown to be capable of differentiating into hair cell like cells [33,39-46]. However, despite great advances, it is apparent that we do not quite understand the molecular basis of hair cell development *in vitro* or *in vivo* or have the ability to control the placement of stem cells and/or hair cell precursors to damaged tissue.

**Figure 1:**
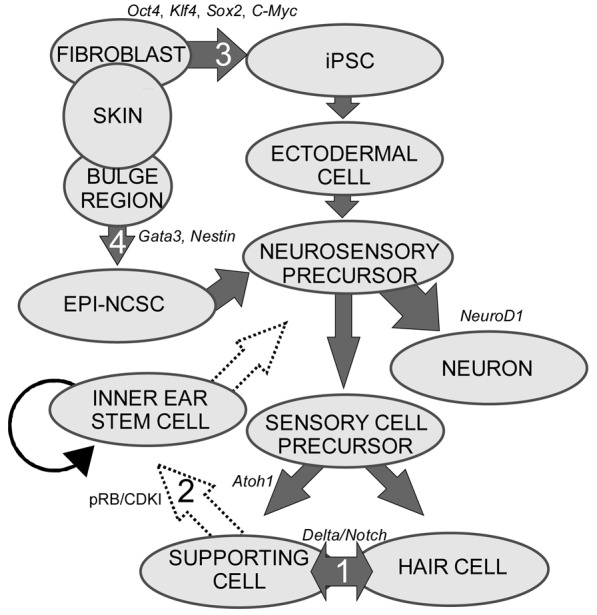
Four methods of regenerating hair cells.

Evolutionarily, the inability of mammalian neurosensory cell re-entry into the cell cycle may be due to the increased cellular diversity and complexity of the mammalian cochlea compared to non-mammalian species, where spontaneous proliferation does occur to restore lost hair cells [4,45,46]. For example, avian systems have two cell types (one supporting cell and one hair cell with graded size change) whereas mammalian cochleae have two distinct hair cell types (inner and outer) in a unique topographic distribution and at least six morphologically distinct supporting cell types in topographic restricted distribution in the organ of Corti [2]. Molecularly, a number of changes make cell re-entry difficult. In mice, hair cell precursors exit the cell cycle in an apex to base gradient from embryonic day 12.5 (E12.5) to E14.5 [47,48]. After cell cycle exit, re-entry is permanently prohibited by an increased concentration of cell cycle inhibitors, including p27^Kip1^ (now CDKN1) [49], an increased phosphorylation burden of the tumor suppressor retinoblastoma (pRB) [50], and a corresponding relative decrease in proto-oncogene (including *Myc*) levels. This evolutionary loss of proliferation may have evolved to protect the delicate inner ear from additional proliferation which would disrupt the existing stereotyped organization and has been shown to be impossible without causing cell death in adult hair cells [27,51]. In mitotically quiescent hair cell precursors, a bidirectional wave of *Atoh1*, originating in the middle-base portion of the cochlea [52], specifies hair cell fate [53-57] and stabilizes the cell fate of surrounding cells to differentiate as supporting cells [58-60] (Figure 1).

Non-mammalian transdifferentiation and spontaneous cell cycle re-entry are seemingly incompatible with the mammalian system as far we understand it. Ideally, differentiated mammalian cells can be forced to re-enter the cell cycle. Forced expansion of proliferation through manipulation of both pRB and especially p27^Kip1^ lasts until P14 in mice but human restoration is needed much later than P14 mouse equivalent. Furthermore, hair cells formed during this time are not viable. While cell specification [47-49,52-57] may render cells incapable of cell cycle entry by changing their molecular signature and enacting epigenetic changes [61-64], inhibiting tumor suppressors and increasing endogenous levels of proto-oncogene may reinitiate the cell cycle for the generation of new hair cells and supporting cells. Important to this is a greater understanding of *Myc*, implicated in both hair cell precursor proliferation and stem cell maintenance [65-69]. Clearly, a more complete understanding of proliferative control in the mammalian ear, including the regulatory network of miRs, may provide insights into the ability of neurosensory cells to re-enter the cell cycle. If the cell cycle could be reinitiated in adult and senescent ears to form either an asymmetrically dividing supporting cell or a symmetrically dividing inner ear stem cell, this could provide ample precursors available for transdifferentiation and/or cell fate re-specification (Figure 1). Until we possess the ability to restart proliferation in the adult ear, transplantation of stem cells seems to provide the sole avenue to hair cell regeneration and for enhancing our understanding of the molecular steps to turn a naïve cell into a hair cell. Some reports suggest the presence of an inner ear stem cell in mammals but the usefulness or potential for regeneration of these cells have not been studied. While a stem cell population in the ear would provide the ideal medium to manipulate hearing restoration, the same principles that would be applied to an ear specific stem cell population can be used with exogenous stem cells for the same purpose. Indeed, lessons learned through manipulation of stem cells *in vitro* may prove useful for the manipulation of ear stem cells *in vivo*. In conclusion, the apparent inability for adult mammalian neurosensory cells to re-enter the cycle makes stem cells the only viable therapeutic option for patients with SNHL.

In summary, both replacement of the entire organ of Corti and the replacement of lost hair cells on a one-by-one basis require the use of stem cells as adult mammalian inner ear neurosensory cells cannot be forced to re-enter the cell cycle as far as we understand it currently. As a result, the potential use of stem cells as therapy for hearing loss has blossomed [1,70-73]. Because of the varied cell types necessary for permanent hearing loss restoration (i.e. inner ear hair cells, sensory neurons), using either multipotent or pluripotent stem cells transplanted to the cochlea followed by incremental inductive cues provides a reasonable therapeutic option. Further progress to a perfect and permanent cure demands first, an understanding of stem cell sources and differentiation potentials; second, a comprehensive knowledge of the minimally essential genes required for ear development, organ of Corti formation, and hair cell maintenance; and last, the technological savvy to safely and non-invasively introduce stem cells, their differentiated derivatives, and/or the appropriate genetic therapy into a damaged cochlea.

## 2. Stem Cells

**Figure 2:**
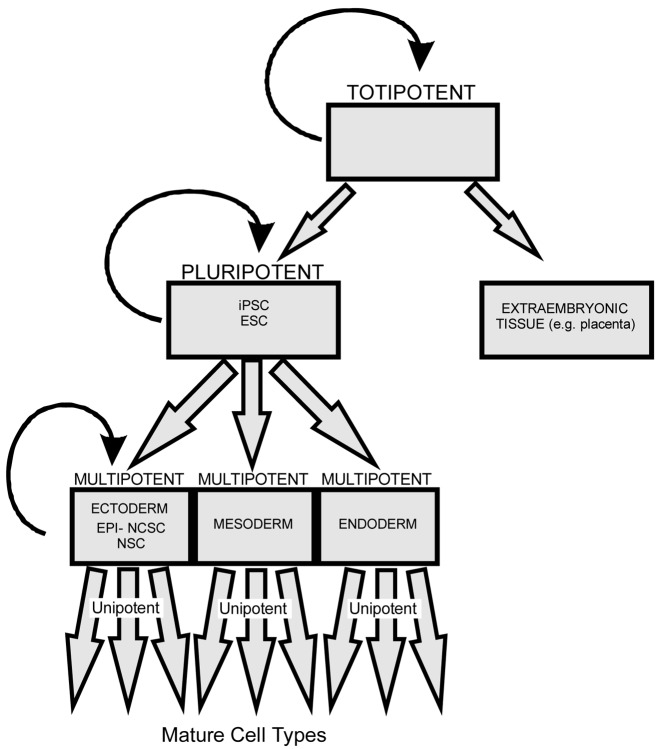
Stem cell differentiation potential.

Stem cells are self-renewing cells with variable differentiation potentials capable of both symmetric and asymmetric division. Stem cells are categorically divided into four groups depending on their potency: unipotent, multipotent, pluripotent, totipotent (Figure 2). The **potency**, or ability of a stem cell to differentiate into a specific cell type, is a property of intracellular gene regulation, epigenetics, and contextual interactions (the so-called stem cell niche) [74-80]. Stem cells exist throughout the body, but the skin, with both hair follicles and fibroblasts, are an **easily accessible and non-invasive source** of both multipotent and pluripotent stem cells. Depending on the potency of the stem cell, which ranges from the ability to make every cell type (totipotent) to the ability to make just a single cell type (unipotent), different stepwise **cell fate restricting** decisions must be used to direct the differentiation of the stem cell into a specific functional cell. Transplanted stem cells can survive in the cochlea to varying degrees but the cell type it differentiates into depends on the type of stem cell it originated from and the molecular signals it receives [81]. Directing the cell fate of stem cells requires knowledge of regulation of the cell cycle and the genes needed for specification of the desired tissue [1,39]. For example, a pluripotent stem cell (such as an iPSC) must first be stepwise restricted to an ectodermal fate, then directed toward an inner ear neurosensory cell fate, and finally to a hair cell fate. In contrast, an ectodermal multipotent stem cell (such as EPI-NCSC) need only be restricted to a neurosensory cell fate and then to a hair cell fate (Figure 1). Obviously, fewer local regulations (as seems to be the case in postnatal ears) [82] driving the direction of differentiation must be substituted by more specification prior to injection of cells to ensure that hair cells differentiate in the right type at the right place.

### 2.1. Unipotent Stem Cells: A One-Way Road

**Unipotent** stem cells have the ability to differentiate into one cell type (or maximally two or three and then are termed bi- or tri-potent). Unipotent stem cells are present in nearly all tissue and primarily function to repair or replace aging or damaged tissue [83]. Unipotent stem cells are often considered progenitor cells [84] and conform to Hayflick's limit which states that after 50-70 population doublings, the progenitor cells undergo apoptosis [84,85]. Unipotent stem cells do not respond to inductive cues along other lineages [84]. While unipotent stem cells are found throughout the body, their inability to differentiate into multiple cell types limits unipotent cells' therapeutic usefulness.

### 2.2. Multipotent Stem Cells: A Bulge of Hope

**Multipotent** stem cells have the ability to differentiate into multiple related cell types. In 2006, it was proposed to use the bulge region of hair follicles as a source of multipotent stem cells [60] as these epidermal neural crest stem cells (EPI-NCSC) [86-88] are capable of differentiating to cells of neural crest origin [89-91]. Using EPI-NCSCs, differentiation into a number of cells of ectodermal lineage has been accomplished [86,89,92], specifically neurons, where a proof of principle experiment was shown to repair a spinal cord injury [93]. Importantly to hair cell therapy, the bulge region is *Gata3* positive (Figure 1). *Gata3* is essential for neurosensory development [94,95], most likely by defining a threshold within which other transcription factors operate to achieve their specific functions. Another multipotent and ectodermally defined stem cell population, neural stem cells (NSCs) have been shown to express hair cell markers when transplanted into the scala tympani [96-100]. NSCs have been isolated from the cochlear nucleus [101], providing a potential source of stem cells to the inner ear. Together, the accessibility and differentiation potential of multipotent stem cells provides a realistic and exciting avenue towards regenerative medicine for patients with hearing loss.

### 2.3. Pluripotent Stem Cells: ESCs and iPSCs

**Pluripotent** stem cells retain the ability to differentiate into nearly all tissue, including any of the three germ layers: ectoderm, endoderm, and mesoderm. Recent reprogramming of mouse embryonic fibroblasts (MEFs) into inducible Pluripotent Stem Cells (iPSCs) with the transcription factors *Oct-4*, *Sox-2*, *Klf-4*, and *C-Myc* [1,102-105] have provided an abundant and ethical alternative to embryonic stem cells (ESCs) [106,107]. Since the reprogramming technique was originally detailed in 2006 [107], a number of changes have made the induction more efficient, safer, and closer to clinical use [102,104,106,108-114]. ESCs are another example of pluripotent stem cells and are the focus of ethical debates due to their tremendous differentiation potential [115-117]. While possessing great differentiation capability, ESCs are susceptible to immune system rejection [118] whereas iPSCs were believed to be accepted [119,120]. However, recent findings show some immunogenicity of iPSCs, possibly due to expression changes due to the *in vitro* conditions they are kept in [121]. Both iPSCs and ESC transplanted in the cochlea differentiate into native tissue but not as efficiently as multipotent stem cells. ESCs transplanted into the spiral ganglion expressed neural markers [122-127] but are unable to express hair cell markers [81,128,129]. In general, pluripotent stem cells perform worse in the cochlea compared to multipotent stem cells. This decreased survival and efficiency is likely due to context [81,97] as the molecular signature is different between ESCs and iPSCs than their counterpart cells of the developing embryo [130] and the fact that less naïve/more differentiated stem cells (i.e. neural stem cells) have differentiated along the same developmental pathway.

**Figure 3:**
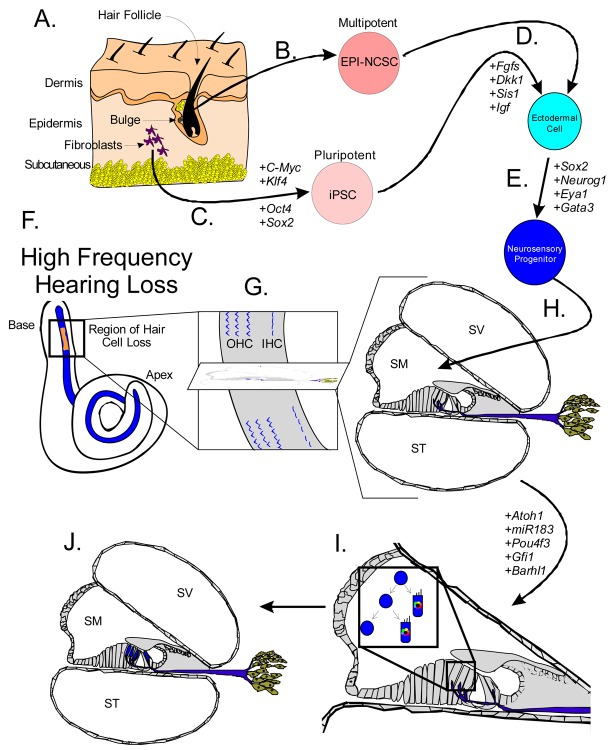
Multipotent stem cells (EPI-NCSCs) and pluripotent stem cells (iPSCs) can be derived from donor skin (A). EPI-NCSCs from the bulge region of the hair follicle (B) and iPSCs formed from fibroblasts using *C-Myc*, *Klf4*, *Oct4*, and *Sox2* (C) can be subsequently restricted in their cell fate to first an ectodermal cell (D) and then a neurosensory progenitor cell (E). The *Fgfs*, *Dkk1*, *Sis1* and *Igf* are among the transcription factors experimentally shown to be important to ectodermal fate [39]. *Sox2*, *Neurog1*, *Eya1* and *Gata3* are important in defining a neurosensory cell. These neurosensory cells are primed to become neurons, hair cells, or supporting cells. Patients with high frequency hearing loss have damaged neurosensory epithelia near the base of the cochlea (F) which often results in the loss of hair cells, marked by a flat organ of Corti (G). In patients with short-term hearing loss, sensory neurons survive (blue) and replacement of hair cells alone may be sufficient treatment. Patients with long-term hearing loss will have a dedifferentiated organ of Corti and a loss of sensory neurons making regeneration of the entire organ of Corti the only solution. Transplantation of either stem cells or neurosensory progenitors into the scala media (H) with subsequent targeting to damaged tissue along with upregulation of hair cell specific genes may enable hair cell regeneration (I). Hair cell fate will be defined and subsequently stabilized through expression of *Atoh1*, miR183, *Barhl1*, *Pou4f3* and *Gfi1* (J).

Both iPSCs and EPI-NCSCs are easily accessible from skin tissue and are promising sources to use for hair cell regeneration (Figure 1 and Figure 3). When stem cells are transplanted in the cochlea, it is necessary to guide the exogenous stem cell towards a hair cell fate through sequential induction of hair cell signals [39,81]. What factors are **minimally** needed is not yet known, but the current focus in developmental ear biology is on identifying the essential genes involved in ear development: otic placode induction, organ of Corti specification, and neurosensory development. Many reviews exist on this topic but we will attempt to briefly cover the essential genes necessary for stem cell applications in the next section.

## 3. Otic Placode Induction, Organ of Corti Specification, and Neurosensory Development Requires a Cornucopia of Genes that Play both Integrated and Independent Functions

### 3.1. Ear Development: Otic Placode Induction and Organ of Corti Specification

A number of genes are essential to each step of ear formation. *Fgf3*, *Fgf8*, *Fgf10* [131-134] and *Wnt1/3a/8a* [135-137] are sufficient as diffusible signals from the hindbrain to induce nearby ectoderm to thicken and form the presumptive otic placode. Induction changes the molecular signature of the placodal region [138-140] and includes the upregulation of *Dlx5*, *Dlx6*, *Eya1*, *Foxg1*, *Foxi1*, *Gata3*, *Gbx2*, *Hes2*, *Hmx2*, *Hmx3*, *Pax2*, *Six1*, *Sox9* and *Spry1* in the preplacode and invaginating placode. Many of these known and other unknown genes are required for axis formation and neurosensory formation as their absence leads to abnormal ear development. Pluripotent stem cells must first be restricted to an otic-ectodermal fate. Among others, signaling of the *Fgfs*, *Dkk1*, *Sis1* and *Igf* have been shown to promote ectodermal fate while inhibiting both endodermal and mesodermal fates [39] (Figure 3). Importantly, cochlear duct elongation and patterning of the organ of Corti are dependent on *Sox2* [141], *Jagged1* [58], *Gata3* [95,132,142], *Lmx1a* [143], *Foxg1* [15,135,144] and *Shh* [145]. Stem cells aimed at forming inner ear hair cells must also be responsive to these signals (Figure 3). After translocation of naïve stem cells to the cochlea, contextual clues may suffice to provide the determination necessary for the differentiation and function of hair cells [104]. However, it is more likely that in the adult, the necessary co-factors are no longer expressed. Therefore, manipulating the above sets of genes is essential to priming stem cells to adopt an organ of Corti prosensory and subsequently a neurosensory cell fate.

### 3.2. Neurosensory Cell Differentiation and Maintenance

**Figure 4:**
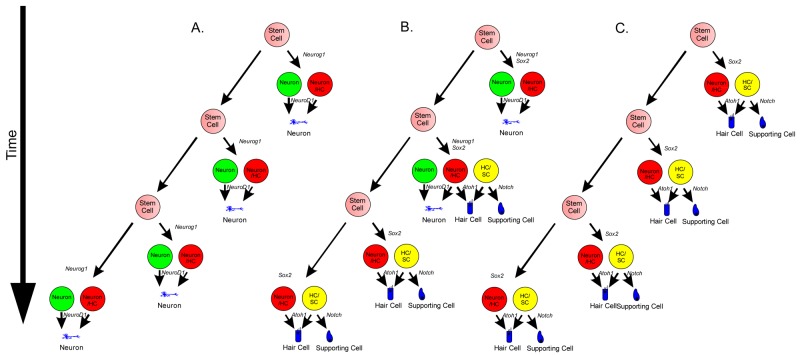
Fate restriction of either an EPI-NCSC or iPSC (pink) yields an additional stem cell (through asymmetric division) and a stem cell/precursor cell which can form a neuron, hair cell, or supporting cell. Clonal and temporal definitions of stem cells in the ear yield unique subsets of differentiated cells. A neuronal precursor (green) will give rise to neurons in the presence of *Neurog1* and *NeuroD1* (A). Neurosensory precursor stem cell populations that will give rise to either neurons and hair cells (red) or hair cells and supporting cells (yellow) are *Neurog1* and *Sox2* positive [60]. In the presence of *NeuroD1*, a neuron is formed. In the absence of *NeuroD1* but in the presence of *Atoh1*, a hair cell is formed. Lastly, in the absence of both *NeuroD1* and *Atoh1*, DELTA/NOTCH signaling stabilizes the supporting cell fate [60]. It is possible that over time, there is a temporal restriction of a stem cell to allow for first a neuronal fate and later hair cell and supporting cell fate (B). Furthermore, a *Sox2* positive cell may only give rise to hair cells and supporting cells but never neurons (C).

Neurosensory cells consist of hair cells, supporting cells, and sensory neurons. Neurosensory precursor populations follow nearly identical cell fate restriction pathways and appear to have a differentiation hierarchy from sensory neuron, to hair cell, and to supporting cell (Figure 1). Furthermore, it is believed that stem cells defined within the ear possess the capability to produce three distinct subsets of cells with either **clonal** [only neurons (Figure 4A) or only hair cell/supporting cells (Figure 4C)] or **temporal** restrictions [initially neurons form and later hair cells/supporting cells form (Figure 4B)] [60]. Terminally differentiated neurosensory cells are the ultimate goal of regenerative medicine.

#### 3.2.1. Sensory Neurons

Sensory neuron formation requires a number of genes that are used to specify the neurosensory domain and differentiate pro-neuronal cells into neurons. Mutations in *Eya1* and *Six1* illustrate the loss of sensory neurons while mutations in *Gata3* and *Shh* result in mice that have a reduction in sensory neurons [95,146,147]. Minimally, sensory neurons are dependent on *Neurog1* [148,149] and *NeuroD1* [56,150-153] as undifferentiated neuronal cells differentiate into hair cells in the absence of *NeuroD1* [56,151] (Figure 1 and Figure 4). Neurotrophins *Bdnf* and *Ntf3* are required for proper guidance and long-term survival of sensory neurons [154-157].

#### 3.2.2. Hair Cells and Supporting Cells

**Figure 5:**
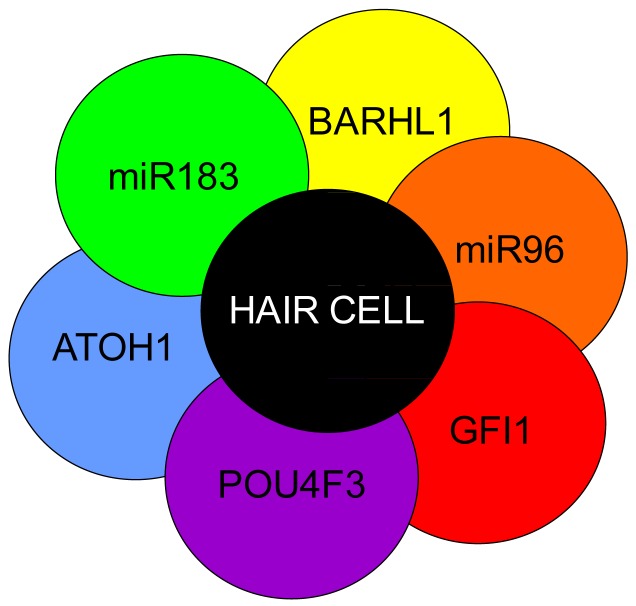
Differentiation of a sensory progenitor cell to a hair cell is dependent on a number of factors.

Hair cell development and maintenance requires a set of genes mostly unique to sensory neurons to facilitate the placement, orientation, and maintenance of hair cells throughout all the sensory epithelia. Formation of hair cells requires proliferation of precursor populations within a specified organ of Corti, differentiation to mature hair cells, and maintenance of hair cells. Precursor proliferation controls the correct number of hair cells and is controlled through regulation of the cell cycle, in part by *Myc* [27,68,69]. After hair cell precursors exit to the post-mitotic state, upregulation of *Atoh1* by E14.5 forces hair cell differentiation [47,158,159]. Lateral inhibition through the DELTA/NOTCH system inhibits hair cell fate for neighboring cells and forces adjacent cells to become supporting cells [15,160-162]. Downstream to *Atoh1* are cell maintenance genes *Pou4f3*, *Gfi1*, *Barhl1* and *miR183* family members [15,163-166] (Figure 5). In the absence of these genes, hair cells and other organ of Corti cells undergo progressive cell death [1,165-168].

## 4. Role of miRs: A Small Name for a Big Player

If transcription factors are the hands on a clock, miRs may well be the gears that really make the clock tick. MiRs are small non-coding 20-25 nucleotide single stranded RNAs that have a two to eight nucleotide “seed” region that interacts with the 3′ end of mature mRNAs. MiRs repress translation of mRNA or degrade mRNA depending on degree of complementarity of the seed region to the target mRNA. MiRs are not fully functional until processed from their immature strands. Abnormalities of processing enzymes, specifically *Dicer* knockouts, have revealed a large impact of miRs (and other small RNAs) on the proper development of the embryo. *Dicer* cleaves the hairpin loop of the pre-miRNA to form the mature miRNA and controls regulatory activity [169]. *Pax2-Cre Dicer* conditional knockouts (CKOs) lose the ability to differentiate into the three germ layers and show defective differentiation [171] and conditional deletion of *Dicer* with *Foxg1-Cre* results in loss of almost all cochlear neurosensory elements [174]. However, *Dicer* also plays other roles making the correlation with miR loss somewhat tenuous. The DICER enzyme is also necessary for siRNA production, an area not well understood in its rapidly growing significance that will not be dealt with here.

### 4.1. miR Control of Stem Cell Self-Renewal

**Figure 6:**
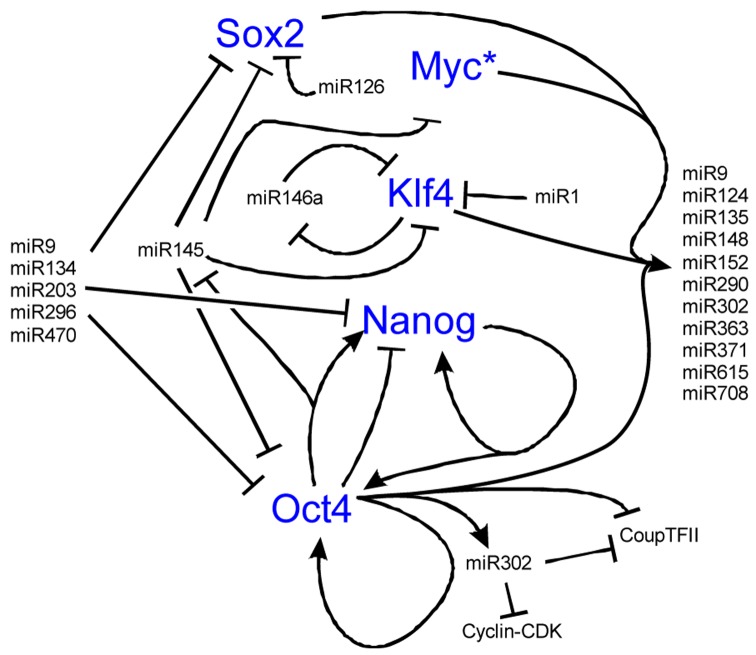
*Oct4*, *Sox2*, *Klf4* and *Myc* are capable of reprogramming of mouse embryonic fibroblasts into pluripotent stem cells.

Both ESCs and iPSCs hold great regenerative potential, but this potential is lost if the stem cell state is lost. A number of genes control the transcriptional regulatory circuit necessary for self-renewal including *Nanog*, *Oct4*, *C-Myc*, *Sox2*, and *Klf4* [175-179] (Figure 6). MiRs are essential to stem cell maintenance and play an important role in stem cell differentiation into hair cells [180]. Throughout the body, miRs are thought to regulate a wide array of genes with a subset of these miRs also expressed in stem cells [180]. Much like the *Dicer* knockouts fail to develop a proper embryo, stem cells deficient in miR processing enzymes do not have proper proliferation or differentiation [181-183]. Identifying which miRs are important is difficult due to a lack of annotation and sheer numbers (over 400 mature miRs in humans) [184,185]. Nonetheless, the four core transcription factors needed for stem cell identity co-occupy 55 distinct transcription units thought to regulate 81 mature miRs [180]. Among these miRs are miR-9, 124, 135, 148/152, 290/371 cluster, 302 cluster, 363 cluster, 615 and 708, all of which show conserved binding [180]. While this may not be overly surprising as the four core transcription factors are thought to co-occupy over 14,000 sites on the human genome [180], it shows the importance of miRs to stem cell control (Figure 6).

**Figure 7:**
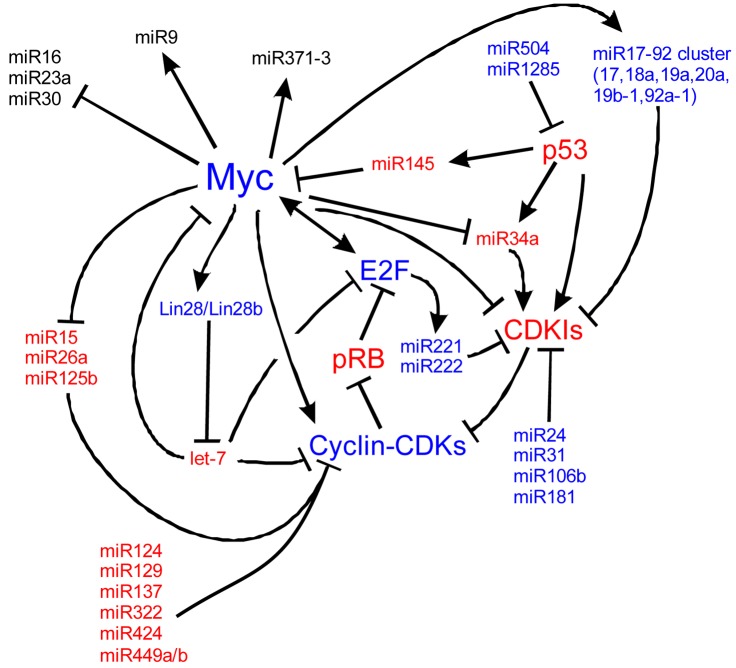
*Myc* is a highly regulated transcription factor that plays roles in stem cell maintenance and proper hair cell formation.

To maintain the stem cell state, the core transcription factors regulate two sets of miRs, the first set that is actively expressed in stem cells, the other set that is silenced in stem cells and that are important in differentiation [180]. Important to this balance is the function of *Myc*, whose inhibition leads to the loss of self-renewal, while activation of *Myc* retains the ability of self-renewal [68,192,193]. *Myc* is both regulated by and controls the regulation of a number of miRs, including miR141, 200, 249 [180,194,195], all important in self-renewal programs. The miRs in the 290-295 and 302 clusters are implicated in cell proliferation and are potential targets of the *Mycs* [196,197]. This suggests that *Myc* is an essential gene highly regulated by miRs to control the balance between self-renewal and differentiation (Figure 7). When the balance shifts away from proliferation, miR activity that was silenced in the stem cell state will repress the stem cell fate to favor the differentiated state (Figure 6 and Figure 7).

### 4.2. miR Control of Stem Cell Differentiation

During differentiation, stem cells must silence their self-renewal program [192]. miRs 9 [198-200], 134, 203, [201-203], 296, and 470 repress *Nanog*, *Oct4*, and *Sox2* [204, 205]. *Oct4* and miR145 create a negative feedback loop that switches stem cells between self-renewal and differentiation [171]. In stem cells, miR145 is low but is highly upregulated upon differentiation and acts to inhibit *Klf4*, *Oct4*, *Sox2* and *Myc* [206]. Exogenous increase of cellular miR145 decreases self-renewal capacity and induces stem cells into an ectodermal cell lineage [171]. Once started along the ectodermal fate, miR124 increases (due to its inhibitor REST decreasing) and leads to the differentiation of progenitor cells to specific cell types, including neurons [207]. In the bulge region of hair follicles in the skin, miR125 (ortholog of lin-4) is critical for exit of stem cells from the cell cycle, and for their differentiation [208-210]. Once cells are differentiated, a number of miRs stabilize this cell fate, including the let-7 family [192,211]. Among the nine-membered let-7 miR family functions is repression of the *Myc* family [211], suggesting one mechanism that maintains the differentiated state and prohibits cell cycle re-entry (Figure 7) [192,212-216]. These examples show the importance of miRs in exiting self-renewal, forced entry into differentiation, and how feedback loops are essential in the control of this process (Figure 6).

### 4.3. miR Control of Hair Cell Differentiation

There are over one hundred miRs present in the cochlea and vestibular systems [172,217,218]. *Dicer* CKOs have shown the importance of miRs (and other small RNAs) to both ear development and hair cell maintenance. *Foxg1-Cre Dicer* CKOs result in loss of almost all cochlear neurosensory elements [174] while *Pax2-Cre Dicer* CKOs [169] and *Pou4f3-Cre Dicer* CKOs [172] show less complete loss.

In vertebrate sensory end organs, miR183 is highly conserved [1,219] and is formed from the same primary transcript as miR96 and 182 [220]. During ear formation, the miR183 family is restricted to hair cells and neurons. Levels of miR183 were characterized in the *Dicer* CKO murine malformed ear and a graded residual expression was seen which correlated to the degree of differentiation seen in the remaining sensory epithelium [169]. Mutations to a miR183 family subunit lead to hearing loss. A mutation in the seed region of miR96 also leads to an autosomal dominant progressive hearing loss [170,220]. These mice have irregular hair cell bundles and have a progressive hair cell loss after birth [221]. In non-mammalian systems, knockdowns of the miR183 family resulted in loss/reduction of hair cells and sensory ganglia [222,223]. Other miRs including miR15, 99, 100, 125, 133 all may play important roles in hair cell development and maintenance [172,173].

“Where there is smoke, there is fire”. This age old idiom holds true for our current knowledge of miRs. While in its infancy, we know miRs play a crucial role in the balance between proliferation and differentiation of the stem cell state and that the miRs are essential for the proper development and maintenance of tissues throughout the body, including the ear. Full understanding of the miRs is required prior to *in vivo* manipulation as deregulation of miRs is implicated in a number of tumors [192,229-232].

## 5. Gene Delivery: Penetrating the Problem

Gene therapy has been achieved in principle in other systems [233-236] but the stereotypic order of cells combined with the complexity of the organ of Corti cellular distribution remains a roadblock in the ear. We have previously discussed the importance of understanding stem cell regulation and what genes are necessary for stem cell differentiation to form functional hair cells, but all this falls on deaf ears unless the stem cells can target the appropriate tissue *in vivo* and the needed genes (i.e. *Atoh1*) can be regulated within the stem cells. Therefore, technical consideration must be given to both the targeting of stem cells to damaged tissue (and their survival) and the regulated gene expression within the stem cells (or *in vivo* newly proliferated cells).

The endolymph can safely accept up to 0.5 μL of stem cells. Stem cells are capable of efficient migration from the point of injection through the entire scala media [40,96,237]. A number of attempts have been made to target damaged tissue in the ear using various stem cells including neural stem cells [96], embryonic stem cells [128,238], and bone-marrow derived stem cells [239,240] but many of these cells were immunorejected or otherwise had unremarkable results [40]. To date, targeting and survival of stem cells remains the limiting factor in applying stem cell therapy to the ear.

Once established in damaged tissue, regulation of gene expression in the stem cell is necessary for tissue restoration. Regulation of stem cells *in vitro* has successfully established hair cell like cells, but manipulation of stem cells *in vivo* has not been accomplished. However, the ear is an ideal playground for viral transduction due to the encasement by the bony mastoid [241]. Three viral vectors are commonly used to target the inner ear tissue: Adenovirus (AV), Adeno-Associated Virus (AAV) and Lentivirus (LV). Each of these three viruses target cochlear cell types. AV targets hair cells, supporting cells, spiral ganglion neurons, fibrocytes, stria vascularis, and Reissner's membrane [40]. AAV targets hair cells, supporting cells, spiral limbus, spiral ganglion neurons, Reissner's membrane, and the spiral ligament [40]. LV targets spiral ganglion neurons, fibrocytes and Reissner's membrane [40]. Each of these vectors have been used to successfully transfect target cells with different genes: GDNF [242], HGF [243], *Catalase* [244], BDNF [245-247], NTF-3 [248], *Atoh1* [249], EGFP [250, 251], and GJB2 [252] in the inner ear. Unfortunately, each of these vectors have shown off-target effects, notably *in vivo* injections of *Atoh1* in the mammalian cochlea have resulted in both normal hair cell formation [158,249,253,254] and *Atoh1* positive hair cells outside the organ of Corti [255]. While viruses do have some tropic specificity, they can target ectopic tissue. Furthermore, viruses themselves may pose a health risk if injecting DNA. The ability to stably and specifically target damaged tissue safely with both exogenous stem cells and the necessary genes is the final step necessary for hearing loss restoration. Multiple laboratories are working on other stem cell targeting and gene regulation expression methods including the use of nanoparticles [256] and other non-viral vectors [257-260] that will be safer, more efficient [261] and will reduce auditory trauma [237,262-264]. For example, regulated expression of target genes could be accomplished through the conditional activation of non-mammalian promoters that drive hair cell specific genes in target tissue, not ectopic sites [265]. Regardless of method, implanted stem cells must be cell fate restricted through regulated gene expression to achieve gene therapy in the ear.

## 6. Conclusion: There is Hope!

With the dawn of gene therapy and technological booms in stem cell technology, regenerative medicine appears to be the wave of the future, promising cures to a wide array of human diseases. While regenerative medicine is still in its infancy and years away from clinical adaptation, it represents new interactions between basic scientists and clinicians. Unprecedented in applicability, regeneration of any tissue requires a solid foundational knowledge of the molecular basis of developmental processes and stem cell regulation. As basic scientists dredge through thousands of genes in the hopes of elucidating the minimal essential gene networks required for induction of directed differentiation and clinicians have increasing numbers of patients asking about permanent cures, the race from bench to bedside has never been more important as well as challenging. Progress in network analysis highlights the controllability of a cell network and the minimal nodes needed to control that network [266].

Millions of patients suffer from hearing loss resulting from lost hair cells in the organ of Corti. Gene therapy and stem cells may offer a permanent cure for afflicted individuals, but the complexity of the ear compared to other systems, makes regeneration of hair cells a daunting task. Yet, the past ten years have shown an incredible advancement in the molecular understanding of ear development [15,267]. A continued improvement in understanding of ear development amalgamated with the nonstop advancements of gene therapy and stem cell biology may someday be fine-tuned music to the ears of many.

## Figures and Tables

**1. Transdifferentiation**. As hair cells differentiate, they express *Atoh1*, and drive the DELTA/NOTCH signaling cascade to force neighboring cells to adopt a supporting cell fate. By inhibiting the NOTCH pathway, it may be possible to change the cell fate from a supporting cell to a hair cell. In mammals, this method is not advisable, unless more cells proliferate. **2. Inner ear stem cells**. A small subpopulation of cycling cells (either endogenous stem cells or made from differentiated supporting cells) will provide a source of cells for directed differentiation and replacement of nearby damaged tissue. These populations have not been well characterized and there is no current technique to restart controlled proliferation giving rise to long-term viable hair cells in the adult in vivo. However, it is hypothesized that by manipulating the balance between proto-oncogenes (*Myc*) and tumor suppressors (pRB and CDKIs), in addition to higher level regulation of tissue specific factors through localization of surface receptors and ligands, re-entry in the cell cycle may be possible. **3. Neural crest stem cells**. *Gata3* and *Nestin* positive multipotent stem cells in the bulge region of hair follicles have the capacity to differentiate into various cell types. *Gata3* is an important gene for the development of the organ of Corti. Selecting proper subpopulations of EPI-NCSCs may provide the necessary foundation to forming novel hair cells. **4. Inducible Pluripotent Stem Cells (iPSCs)**. IPSCs derived from mouse embryonic fibroblasts, through the forced expression of *C-Myc*, *Klf4*, *Sox2*, and *Oct4*, have been important in providing alternative pluripotent cells from embryonic stem cells. From the pluripotent and multipotent state, an ectodermal and neurosensory fate restriction is necessary to form hair cells. Neurosensory cells can form neurons with the expression of both *Neurogenin1* (*Neurog1*) and *NeuroD1* while hair cells form in the absence of *NeuroD1* combined with *Atoh1* upregulation. Both EPI-NCSCs and iPSCs can be derived from the skin of the donor. Adapted from Fritzsch *et al* [60].

All stem cells have the ability to divide symmetrically and asymmetrically. As a stem cell asymmetrically divides it loses the potential to differentiate into cells of other lineage. Totipotent stem cells have the ability to form any cells derived from both pluripotent stem cells and extraembryonic tissue (including the placenta). Pluripotent stem cells, including both iPSCs and embryonic stem cells (ESCs), have the ability to differentiate into all three germ layers (ectoderm, mesoderm, and endoderm). Multipotent stem cells such as EPI-NCSCs and NSCs have less differentiation potential than pluripotent stem cells but both can form multiple cells of related lineage. Unipotent stem cells possess the least differentiation potential and can generate only a limited array of cell types.

*Atoh1* is required for differentiation into hair cells whereas *Pou4f3*, *Gfi1* and *Barhl1* are downstream to *Atoh1* and are important for the maintenance of the hair cells. A number of miRs have been shown to be essential in the formation of hair cells and include the miR183 family (miR96, 182, and 183) as their losses result in hearing loss [1,169-171]. Additionally, miRs 15, 99, 100, 125, 133 may also be important to hair cell development but have not been well characterized [172,173]. Adapted from Pauley *et al* [1].

*Nanog* is known to be important for the maintenance of the stem cell state in a feedback loop with *Oct4*. While there are a number of transcriptional networks essential to the balance between stem cell self-renewal and differentiation, we omit the transcription factors to highlight the tight regulation and feedback loops that miRs play in stem cell control. Over eighty miRs are activated by the key stem cell transcription factors while other miRs are silenced. Most of these miRs have not been well characterized functionally but those that have display both positive and negative feedback loops as illustrated. Notably, miR145 is repressed by *Oct4* to maintain pluripotency but at the onset of differentiation, lower levels of *Oct4* allow miR145 to repress the pluripotent state through repression of *Oct4*, *Sox2*, *Klf4*, and *Myc*. Also note known feedback loops with miR9 and miR146. Among the 81 miRs upregulated by the binding of the four transcription factors is miR9, which after activation represses *Sox2*, *Nanog*, and *Oct4*. MiR146 forms a double negative feedback loop with *Klf4*. Together, the miRs are important to both maintaining the pluripotent state and when the balance is shifted towards differentiation, maintains the differentiated state by inhibiting stem cell transcription factors. Interactive pathways adapted from [114,186-191]. * See Figure 7 for *Myc* regulation.

*Myc* is also implicated in cancers, cell cycle regulation, apoptosis, metabolism, and cellular differentiation. *Myc's* regulation is complex. Here we detail many of the known upstream and downstream miR regulators of *Myc* and the cell cycle. In blue are molecules that are important for proliferation and in red, molecules suppressing proliferation to favor cell cycle exit. At the core of the cell cycle is the tightly regulated G1-S checkpoint which is regulated by the phosphorylation state of retinoblastoma (pRB). When unphosphorylated, pRB binds E2F family members which are then unable to bind S-phase promoting genes. Phosphorylation of pRB releases E2F and the cell is able to enter the S-phase of the cell cycle. *Myc* is a central cog in cell cycle regulation through its signaling of miRs, the cyclins, and its repression of cyclin dependent kinase inhibitors (CDKIs) such as p27^Kip1^. Positive *Myc* regulation of a number of miRs, including the miR17-92 cluster, represses cell cycle inhibition. Negative regulation of other miRs, including miR15, 26a and 125b, de-represses negative regulation of the cell cycle. Other miRs, not currently characterized to be responsive to *Mycs*, are also responsible for cell cycle dynamics. Understanding the regulation of *Myc*, of the miRs and of the cell cycle is essential to manipulating stem cells and ultimately, manipulating these pathways may be the key to forced re-entry of hair cells into the cell cycle. *Myc* pathways adapted from [224-228].
